# Supplementary material for: Dynamic transcriptomic profiles of zebrafish gills in response to zinc supplementation
Source: BMC Genomics. 2010 Oct 11;11:553. doi: 10.1186/1471-2164-11-553 (PMC3091702; doi:10.1186/1471-2164-11-553)
Supplement: Additional file 2 — Interactive Direct Interaction Network representing the molecular interactions between zinc, copper, iron, calcium and proteins encoded by transcripts changed by zinc supplementation. Mini web-site containing index.html and hyperlinked pages in subdirectory describing a Direct Interaction Network automatically generated based on curated interactions contained within the proprietary PathwayArchitect database. Ovals represent proteins and the circles symbolize metal ions. Objects are coloured by their abundance in zebrafish at the time-point they were significantly different from the control is a scale from -4 fold (dark green) to +4 fold (dark red). Where significant differences were found at more than one time-point, the colour overlay shows expression at the first instance. Dark blue squares denote 'binding', and light blue squares 'expression'; green squares stand for 'regulation', green diamonds for 'metabolism', and green circles for 'promoter binding'. Arrow heads indicate directionality of the interaction where annotated. All nodes and edges can be further interrogated by selecting the relative area of the image. [file 1471-2164-11-553-S2.zip › PathwayArchitect Zn xs DIN/635227.html]

# PROTEIN MODIFICATION:

|  |  |
| --- | --- |
| Type | PROTEIN MODIFICATION |
| Effect | None |


---

|  |  |
| --- | --- |
| Score | 0 |


---

|  |  |
| --- | --- |
| Reference Count | 4 |


---

|  |  |
| --- | --- |
| Mechanism | Unknown |


---

|  |  |
| --- | --- |
| Reference:0 || Sentence | "In response to DNA damage or stalled replication, the ATM and ATR protein kinases activate the checkpoint kinases Chk1 and Chk2, which leads to hyperphosphorylation of Cdc25A." |
| PMID | 14603323 |
| Year | 2003 |
| Species | Human |
| Journal | Nature |
| RefScore | 2 |
| Source | PArchNLP |
  |
|  |  |


---

|  |  |
| --- | --- |
 Reference:1 || Sentence | "We show that IR-induced destruction of Cdc25A requires both ATM and the Chk2-mediated phosphorylation of Cdc25A on serine 123." |
| PMID | 11298456 |
| Year | 2001 |
| Species | Mouse |
|  | Human |
| Journal | Nature |
| RefScore | 1 |
| Source | PArchNLP |
  ||  |  |


---

|  |  |
| --- | --- |
 Reference:2 || Sentence | "We also show that tumour-associated Chk2 alleles cannot bind or phosphorylate Cdc25A, and that cells expressing these Chk2 alleles, elevated Cdc25A or a Cdk2 mutant unable to undergo inhibitory phosphorylation (Cdk2AF) fail to inhibit DNA synthesis when irradiated." |
| PMID | 11298456 |
| Year | 2001 |
| Species | Mouse |
|  | Human |
| Journal | Nature |
| RefScore | 1 |
| Source | PArchNLP |
  ||  |  |


---

|  |  |
| --- | --- |
 Reference:3 || Sentence | "In response to ionizing radiation, Cdc25A is phosphorylated by both Chk1 and Chk2 on Ser-123." |
| PMID | 12759351 |
| Year | 2003 |
| Species | Human |
| Journal | J Biol Chem |
| RefScore | 2 |
| Source | PArchNLP |
  |


---

|  |  |
| --- | --- |
